# The Epidemiology of Hepatitis E in Israel and Potential Risk Factors: A Cross-Sectional Population-Based Serological Survey of Hepatitis E Virus in Northern Israel

**DOI:** 10.3390/v17040536

**Published:** 2025-04-07

**Authors:** Rasha Daniel, Shira Zelber-Sagi, Mira Barak, Eli Zuckerman

**Affiliations:** 1 Haifa and Western Galilee Central Laboratories, Clalit Health Services, Nesher 20300, Israel; 2 School of Public Health, Faculty of Social Welfare and Health Sciences, University of Haifa, Haifa 3498838, Israel; shira.zelber@gmail.com; 3 Head of Medical Laboratory Sciences, Zefat Academic College, Safed 13206, Israel; mirabarak@zefat.ac.il; 4 Liver Unit, Carmel Medical Center, Faculty of Medicine, Technion Institute, Haifa 3498838, Israel

**Keywords:** hepatitis E virus, general population, seroprevalence, hot spot analysis

## Abstract

Hepatitis E Virus (HEV) has gained public health attention as one of the causative agents of viral hepatitis. Our study aimed to provide data about HEV seropositivity in the Israeli general population, including its seroprevalence geographical distribution, and to identify variables as possible risk factors for HEV exposure. A seroprevalence cross-sectional study was conducted: HEV serological status was determined in 716 blood samples collected from the routine check-up blood samples. Demographic information was available for all samples. The overall prevalence of HEV IgG in an apparently healthy population in the north of Israel was 10.5%, with no evidence of positive HEV IgM. There was a significant association between HEV seropositivity and elderly age and low socioeconomic status (SES). The age-adjusted seroprevalence was significantly lower among Jews compared to Arabs with a rate ratio of 2.02. We identified clusters (hot spots) of HEV infection in three regions under study. Our results confirmed a high prevalence of anti-HEV in the country where clinical hepatitis E is not endemic. For the first time, this study showed that a hot spot analysis was able to provide new knowledge about actual exposure zones. As HEV infection is not a notifiable disease, it is probably underdiagnosed. Thus, better awareness among physicians is warranted.

## 1. Introduction

Hepatitis E Virus (HEV), a member of the Heperviridae family, is globally classified into eight genotypes (1–8) [1,2]. HEV strains affecting humans are classified into genotypes 1, 2, 3, 4, and, most recently, 7; the other three genotypes can infect animals but are not transmissible to humans. Genotypes 1 (G1) and 2 (G2) are limited to human hosts. Genotypes 3 and 4 (G3 and G4) have multiple hosts and can also be transmitted to humans. Genotypes 5 (G5) and 6 (G6) are known to infect wild boar; however, it is unknown whether these genotypes can be transmitted to humans. Finally, genotypes 7 (G7) and 8 (G8) infect dromedary and Bactrian camels, respectively [1,2,3]. Recently, G7, isolated from a dromedary camel, was also associated with chronic viral hepatitis in a transplant recipient [4].

HEV is mainly feco-orally transmitted, usually by contaminated drinking water. It can also be transmitted through food contaminated with HEV or via animal-to-human transmission through feces or direct contact. Other possible modes of transmission such as parenteral, human blood supply, and vertical transmission from mother to child have been reported [3,5,6,7]. HEV usually causes an acute, self-limiting infection with non-existent/mild symptoms that can evolve even to fulminant hepatitis; infections can also induce acute liver failure in pregnant women. Over the past decade, there have been new aspects associated with HEV infection, mainly the possibility of chronic hepatitis caused by G3 in immunosuppressed individuals [8,9,10]. In developing countries, the epidemiology of HEV seroprevalence among the general population varies extensively; the wide range of results appears to depend on many variables, including the serological assay used, the geographical region, and the study cohort [11]. Recently, a meta-analysis study aiming to assess global HEV seroprevalence estimated that 12.47% of the global population have experienced past infection of HEV based on the anti-HEV IgG antibody. Africa and Asia have been previously recognized for the high prevalence of HEV [12]. An epidemiological study from Germany found an overall HEV seroprevalence of 16.8% among adults in Germany, and it was determined that seroprevalence increased with age [13]. Similarly, high seroprevalence was found in blood donors in Denmark (20.6%) and southwestern England (16%) [14,15]. Related to recent European reports, some data have shown that blood donors’ HEV seroprevalence rates are higher than those reported in patients with acute hepatitis [16]. In addition to this high prevalence in blood donors, transmission of HEV by blood transfusion has also been observed [17].

In the last 20 years, only one HEV seroprevalence study has been performed among the Israeli general population. The study was performed during 2009–2010 by the Israeli Center for Disease Control (ICDC) in the central area of Israel, using anonymous diagnostic laboratories’ residual samples and healthy blood donor samples stored at the national serum bank of the ICDC. The study found an overall prevalence of 10.6% HEV IgG, as well as a significant association between HEV seropositivity and advanced age, low socioeconomic status (SES), Arab ethnicity, and being born in Asia, Africa, or the former Soviet Union [18]. HEV was also found to be circulating in environmental sewage samples in Israel. Most of the positive samples were identified in the region of Haifa, in the northwest area of Israel [19]. Subsequently, two additional studies showed a high HEV IgG prevalence among swine and camels in Israel [20,21]. One of the most effective methods for investigating prevalence is hot spot analysis.

Aiming to address epidemiological gaps in our understanding of HEV as an emerging pathogen that contributes to public health concerns, we investigated HEV seroprevalence in the “apparently healthy population” in North Israel, identified potential risk factors for seropositivity, and looked for the relationship between geographic locations of features and HEV seroprevalence. One of the most effective methods for investigating prevalence is hot spot analysis. While a basic comparison of seroprevalence across cities or villages provides a general overview, it does not account for spatial dependencies or clustering effects, treating each location as an isolated unit and potentially overlooking important epidemiological patterns. To address these limitations, the present study incorporates hot spot analysis to identify statistically significant clusters of high and low seroprevalence. This approach enables the detection of spatial trends that may be influenced by underlying factors such as population density, mobility patterns, healthcare accessibility, and environmental conditions.

## 2. Methods

### 2.1. Study Population

The study was a cross-sectional seroprevalence study based on the probability sampling of retrospective routine blood examinations from an “apparently healthy population”. This population included individuals who are generally healthy without any underlying medical conditions according to their medical records, who undergo regular annual blood tests as part of a “check-up” procedure in the department of routine chemistry in the Central Laboratory of Clalit Health Services (CHS) in the Haifa and Western Galilee district. CHS is the largest and leading healthcare organization in Israel, serving 52% of the Israeli population across 1500 clinics and throughout 200 municipalities. The Haifa and Western Galilee district is one of the nine districts served by CHS and is considered the largest in Israel, serving almost 800,000 people from both Jewish and Arab populations and receiving about 5000 tests per day for routine chemistry check-ups. In order to ensure a sample representative of both the Jewish and Arab populations and the different age groups, proportional stratified sampling was used. During the year 2017, 50 blood samples from each stratum were selected randomly each day from the routine check-up blood sample results list. If not ordered initially, the samples were further tested for HCV Ab, HBs Ag, and hepatitis B core Ab (HBc Ab), in addition to conducting the main liver function profile tests (aspartate transaminase (AST), alanine transaminase (ALT), gamma-glutamyl transferase (γ-GT), alkaline phosphatase (ALP), albumin, and bilirubin). Only sera from individuals over the age of 18 years and without laboratory evidence of chronic or acute liver disease were included in the study. Demographic information (age, gender, population group, residency) was collected for each sample using CHS Auto-Reports software (https://autoreport.info/). SES was assessed by socioeconomic rank, as defined by the Israel Central Bureau of Statistics 2017, calculated using multiple sociodemographic and economic factors, including the financial resources of the residents, their housing conditions, their motorization level, and their education and employment profile [22]. Ranks ranged on a scale from one to ten, with lower ranks representing a lower SES.

### 2.2. HEV Laboratory Diagnosis

All serum samples were tested once for HEV IgG using a DS EIA-ANTI-HEV G kit from Diagnostics Systems (Diagnostic Systems Italy, Saronno, Italy). HEV IgG-confirmed-positive samples were tested for HEV IgM with a DS EIA-ANTI-HEV M kit from Diagnostics Systems (Diagnostic Systems Italy, Saronno, Italy). Positive samples were re-tested in duplicate. Samples that were reactive two or three times were reported as seropositive.

Both IgG and IgM kits are based on Recombinant ORF2 and ORF3 antigen coating and capable of detecting antibodies against 4 HEV genotypes (1–4). According to the manufacturer, the relative sensitivity and specificity of both assays are 100%. An earlier evaluation study conducted by the U.S. Centers for Disease Control and Prevention (CDC) of 6 serologic assays for IgM antibodies against HEV identified the assay manufactured by Diagnostics Systems as having the best performance in terms of diagnostic sensitivity and specificity of 95.2% and 98% [23].

### 2.3. Hot Spot Analysis

In order to depict the relationship between the geographic locations and HEV seroprevalence in the Haifa and Western Galilee area, we employed an approach of spatial autocorrelation and cluster mapping: Getis-Ord hot spot analysis [24] using ArcMap software (version 10.6.1) [25]. According to this approach, a hot spot is a cluster of data with a high attribute value surrounded by lower-value data. An area is considered to be a statistically significant hot spot when the sum of the attributed data of neighboring features is different from the expected sum if the data were randomly divided over the space. When the found difference is too large to be the result of random chance, a very low *p*-value is present, indicating the statistical significance of the cluster. Clusters of significantly high values are considered hot spots, while clusters of low values are considered cold spots. During the data collection and processing, all study sites (cities or villages) were given geographical coordinates based on the government mapping website and were manually checked for (typing) errors. A further selection criterion was applied before the data were analyzed: only sites that had more than two collected samples were included in the final analysis.

In order to reflect any type of false discovery rate (FDR) correlation, we calculated the Gi-Bin field to identify statistically significant hot spots. Features in the +/−3 bins reflect statistical significance with a 99% confidence level, features in the +/−2 bins reflect a 95% confidence level, features in the +/−1 bins reflect a 90% confidence level, and the clustering for features in bin 0 do not form a statistically significant hot or cold spot regardless of whether or not the FDR correction is applied. Furthermore, to reject the assumption that close points are affected by the same phenomenon more strongly and to demonstrate that it is not the geographical proximity that makes certain hot spots more exceptional than the other examined locations (rather, it is the high prevalence of HEV IgG findings), we used the inverse distance weighting (IDW) method [26]. The IDW method assumes that each measured point has a local influence that diminishes with distance and gives greater weight to the points closer to the prediction location than to those farther away. The spatial distribution was also normalized for age to improve the identification of hot spots through the “mining” of spatial patterns. The mean age for each study site was entered as a weight factor.

### 2.4. Statistical Analysis

Data were analyzed using SAS version 9.4 (SAS Institute, Cary, NC, USA). *p*-Values of 5% were considered statistically significant. Categorical data were reported as frequencies and percentages. The overall HEV IgG seroprevalence was standardized (per 100) to age, sex, and ethnicity according to the direct method using the 2017 Israel standard population from the Central Bureau Of Statistics “lamas” (age groups of 15–19, 20–24, 25–34, 35–44, 45–54, 55–64, 65–74, 75). In order to explore the associations between study characteristic variables (sex, age, ethnicity, living status, and socioeconomic status) and HEV IgG, we used logistic regression models (a univariable model for each variable and a multivariable model that included all study characteristic variables in the same model). The sample size for each group was determined based on the expected prevalence of HEV antibodies according to similar data in previous studies from Israel and was calculated using the “sample size for proportions”. As commonly accepted, two-sided significance was determined as 5%, and a power of 80% was used for the calculations.

### 2.5. Ethical Aspects

The study was performed in accordance with the approval of the Helsinki declaration by both CHS (approval no. 0177-15-COM) and the University of Haifa ethics committee (approval no. 028/19).

## 3. Results

### 3.1. Description of the Study Population

Table 1 represents the demographic characteristics of all participants including sex, age, ethnicity, place of residence, and socioeconomic status. Overall, 716 blood samples were collected: 42.5% were men, the age range was from 18 to 80 years, and the mean age was 44.3 ± 17.5.

Of the 716 study samples, 46.8% were obtained from Arabs. According to data available at the Israeli Central Bureau of Statistics, the Arab population constitutes about 20% of the Israeli population. The present study included oversampling of the Arab group (46.8% vs. 20%) to be able to analyze the data in this relatively small group and to ensure a good representation of the Arab population. However, the oversamples were statistically adjusted in the final results so that this group is represented in proportion to its actual share of the Israeli population.

### 3.2. HEV Seroprevalence Results

Table 2 and Table 3 summarize the crude and standardized rate ratios in the different groups. Of 716 samples, 75 tested positive for HEV IgG 10.5 [8.2–12.7] with no evidence of positive HEV IgM, indicating past HEV infection. The overall HEV IgG seroprevalence standardized to age, sex, and ethnicity was 9.8 [6.9–12.8]. The overall HEV IgG seroprevalence standardized to age, sex, and ethnicity separately was 11.9 [9.2–14.6], 10.4 [8.3–12.8], and 9.4 [6.9–11.9], respectively. The age-adjusted seroprevalence in the Jewish group was 8.6 [5.7–11.6] and in the Arab group 17.5 [11.9–23.1]. Comparing the age-adjusted seroprevalence between the Arab and Jewish groups yielded a significant difference with a rate ratio of 2.02 (95% CI 1.26–3.22, *p* < 0.001). Comparing the age-standardized rate between females (12.6 [8.9–16.3]) and males (10.9 [6.9–14.8]) yielded no significant difference.

Table 4 show the associations between study characteristic variables and HEV IgG. Looking for potential risk factors, seropositivity was significantly associated with elderly age (OR = 59.05, 95%CI 21.1–165.1, *p* = 0.001) and low SES (OR = 0.41, 95%CI 0.21–0.82, *p* = 0.01). Within the different age groups, a significant gradual increase in the HEV seroprevalence rate was observed from the youngest to the oldest subjects. The odds of being anti-HEV-positive were higher in middle-aged individuals (aged 55–74) and in elderly individuals (aged 75 years and above) compared with individuals in the younger group (18–34 y/o). Anti-HEV IgG positivity in females and in the Arab population group was higher compared to males and the Jewish population group accordingly, but these differences were not significant.

### 3.3. Spatial Analysis

**Figure 1:**
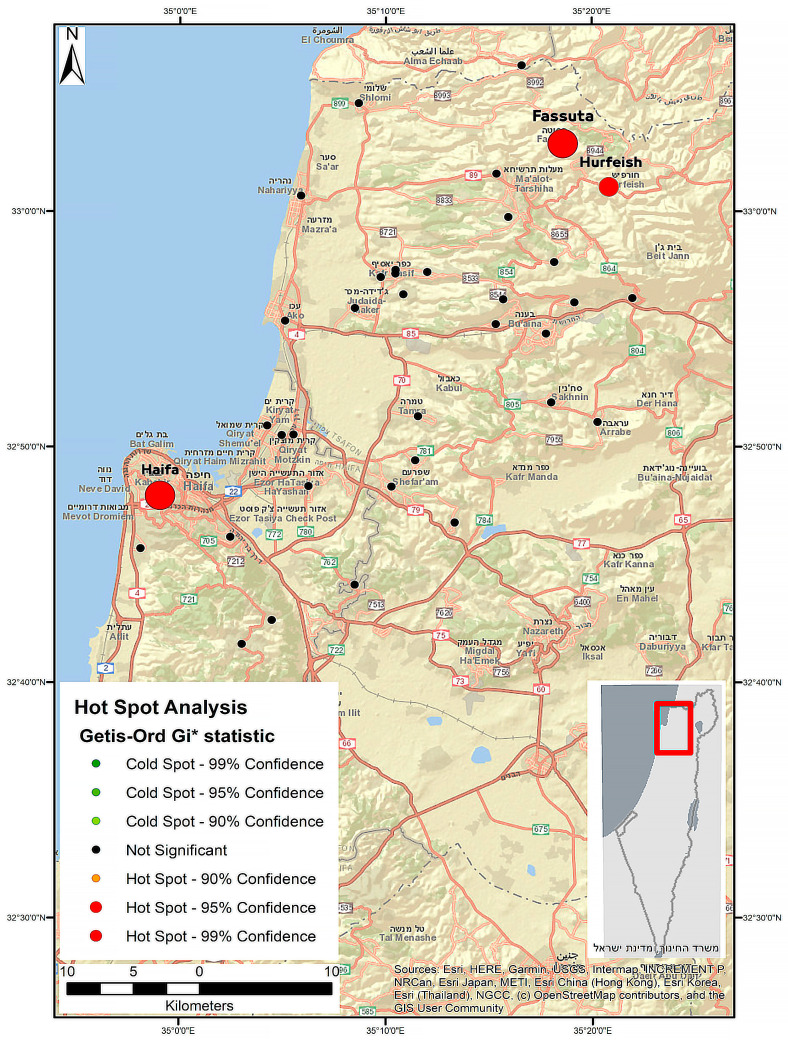
Hot spot classification by applying the Getis-Ord Gi* approach in the study area. Each circle represents a region, and the larger the circle, the higher the seroprevalence. The circle color represents the Gi* *p*-value ranges.

Figure 1 displays the HEV IgG hot spot classification in the study area, conducted using the Getis-Ord Gi* approach implemented via ArcMap. According to the hot spot analysis, three major sites were identified as hot spots for HEV IgG prevalence with a 99% confidence level (Haifa, Fassuta, and Hurfeish). 

## 4. Discussion

This study presents a cross-sectional assessment of the HEV antibody prevalence among a representative sample of an apparently healthy population from northern Israel. Our overall prevalence (10.5%) is concordant with a previous seroprevalence study (10.6%) that used the same serological assays and analyzed healthy blood donors and anonymous diagnostic blood samples from people living in the central region of Israel. However, there was a difference in the calculated age-adjusted rate in our study (11.9%) compared with the previous one (7.6%) [18]. The potential reasons for the difference in the rates may be attributed to the different representative samples for the population used in the studies. The outcome of the previous study was based on healthy blood donors and anonymous diagnostic blood samples—two groups that are not necessarily representative of the general Israeli population. Sampling blood donors may underestimate the real prevalence in a selectively healthier population. Furthermore, there was a poor representation of the Arab population in the previous study compared to our study. However, our sample was also adjusted for ethnicity, but still the age-adjusted rate within the Jewish group (8.6%) was higher than the overall age-adjusted prevalence in the previous study (7.6%).

The higher age-adjusted rate observed in our study may also stem from the different geographical regions that each study represents—the north of Israel in the current study versus the central region of Israel in the previous study. Northern parts of Israel are more exposed to HEV G3, which is possibly circulating in the country and was recently identified in local sewage facilities mainly in the north [19]. Furthermore, most of the swine slaughtering and breeding farms are located in the north of the country. Outside of Israel, HEV G3 strains were also recently detected in sewage and in environmental water samples in Germany [27] and France [28]. Three clusters (hot spots) of HEV infection were identified in three regions under this study: Haifa, Fassuta, and Hurfeish. The high HEV seropositivity rate observed in the Haifa region is consistent with the findings recently observed in a study aimed at investigating the occurrence of HEV infection in Israel through molecular screening of raw sewage samples [19]. According to the study, 14 of 160 sewage samples tested were positive for HEV RNA, with most of them identified in the Haifa region. Sequence analysis revealed HEV G3 sequences in these RNA-positive samples.

It is well known that swine are the main reservoir of HEV worldwide and that the virus is present on most swine farms [5,29,30,31]. A recent pilot study in Israel aiming to assess the status of HEV infection among swine farmers showed that domestic swine in Israel are infected with HEV G3 and three-quarters of all tested swine are anti-HEV-seropositive [20]. Fassuta is a local council in the north of Israel and all of its inhabitants are Christian. The Christian religion permits the eating of swine, and many members of the Christian community in Israel do eat it. Otherwise, swine production and breeding are limited in Israel, mainly located in communities with a significant Christian population. Fassuta is one such community. Our main hypothesis is that the high HEV seroprevalence in Fassuta is a result of zoonotic infection transmitted by swine and can be attributed to exposure to HEV G3, which is possibly circulating in this area. The higher HEV seroprevalence observed by a hot spot analysis in Hurfeish may be associated with its overall lower SES and poor sanitary conditions, which make HEV infection more likely. Hurfeish is a rural town categorized in socioeconomic cluster 3–4 out of 10 according to the Central Bureau of Statistics in Israel. This places it in a lower range of SES and it falls behind in terms of economic opportunities, infrastructure, and diversity. However, HEV G1 or G2 infections caused by contaminated water are linked to lower SES. As in Israel, no outbreak of HEV G1 or G2 infection had been reported so far. Our hypothesis regarding the association between higher HEV seroprevalence and the lower SES in Hurfeish must be supported by evidence of HEV G1 or G2 infection or by the presence of HEV G1 or G2 RNA in the sewage of Hurfeish. However, the cause of the high HEV seroprevalence in Hurfeish remains unknown.

The increase in HEV seroprevalence with age found in the present study is consistent with reports from other countries [8]. We believe that the increase in seropositivity with age seen here may be attributed to a cohort effect or cumulative exposure. Our thought is supported by the fact that one of the main sources of HEV transmission is through the fecal–oral route, and improvements in sanitation and general living conditions can explain the decreasing rate of HEV transmission over time in the youngest groups and the cohort effect. The association of HEV exposure with lower SES is in agreement with other studies worldwide, which found that low SES is a major risk factor for increased prevalence of HEV infection [5,8]. Another risk factor associated with HEV seropositivity is ethnicity. Our study included oversampling of the Arab group (46.8% vs. 20%) in order to analyze the data in this relatively small group and to ensure a good representation of this population. However, the oversampling was statistically adjusted so that the group was represented in proportion to its actual percentage of the Israeli population. The overall calculated ethnicity-adjusted rate was 9.4%, and the age-adjusted seroprevalence was lower among Jews compared to Arabs. In our study, there was a strong correlation between ethnic categories and living residence. Within the Arab population, 78% live in a village compared to 14% of the Jewish population. We believe that the higher prevalence in the Arab population may be associated with lower level of sanitation and SES that are characteristic of their living areas.

Overall, the results of the present study confirm a high prevalence of anti-HEV in the country where clinical hepatitis E is not endemic with evidence of autochthonous infections and demonstrate that HEV circulates in the north district of Israel. Our results emphasize the importance of increasing the awareness of HEV infection among physicians and the necessity of establishing an algorithm for HEV diagnosis and screening in different Israeli populations.

However, this study has several limitations to consider. Being a cross-sectional study, a temporal association cannot be determined, and, thus, no causal inference can be made. To detect the seroprevalence in the different ethnic groups and to identify if these specific population groups are at high risk of HEV seroprevalence, samples were taken from within the Haifa and Western Galilee region, which is characterized by mixed ethnic populations. However, lack of representation of similar populations from other parts of Israel (center and south) decreases the external validity of our results. Further studies are necessary to define the clinical and epidemiologic burden of HEV infection in this area and to identify additional risk factors for HEV infection.

## Figures and Tables

**Table 1:** Characteristics of the study population.

Variables		Total Sample N = 716
Sex	Male	304 (42.5)
	Female	412 (57.5)
Age (years)	18–34	260 (36.3)
	35–54	263 (36.7)
	55–74	145 (20.3)
	75+	48 (6.7)
Ethnicity	Arab	335 (46.8)
	Jewish	381 (53.2)
Place of residence	Village	314 (43.8)
	City	402 (56.2)
Socioeconomic status	Low rank 1–3	184 (25.7)
	Intermediate, high rank 4–10	532 (74.3)

**Table 2:** Age-, sex-, and ethnicity-standardized and overall HEV IgG seroprevalence.

All	Standardized Rate (per 100) 95% CI
Standardized by age, sex, and ethnicity group ^1^	9.8 (6.9–12.8)
Standardized by age ^2^	11.9 (9.2–14.6)
Standardized by sex ^3^	10.4 (8.3–12.8)
Standardized by ethnicity ^4^	9.4 (6.9–11.9)

^1^ Age-, sex-, and ethnicity-adjusted seroprevalence in the Israeli standard population as in “lamas”, 2017. ^2^ Age-adjusted seroprevalence in the Israeli standard population as in “lamas”, 2017 (15–19, 20–24, 25–34, 35–44, 45–54, 55–64, 65–74, 75+). ^3^ Sex-adjusted seroprevalence in the Israeli standard population as in “lamas”, 2017. ^4^ Ethnicity-adjusted seroprevalence in the Israeli standard population as in “lamas”, 2017.

**Table 3:** Age-standardized, male/female, and Arab/Jewish HEV IgG seroprevalence.

	Crude Rate (per 100) 95% CI	Standardized Rate ^1^ (per 100) 95%CI	Standardized Rate Ratio 95% CI
All	10.4 (8.5–13.0)	11.9 (9.2–14.6)	**-**
Female	10.9 (8.3–14.4)	12.6 (8. 9–16.3)	1.16 (0.73–1.85)
Male ^2^	9.8 (6.9–13.7)	10.9 (6.9–14.8)	1
Arabs	12.5 (9.4–16.6)	17.5 (11.9–23.1)	2.02 (1.26–3.22)
Jews ^2^	8.6 (6.3–12.0)	8.6 (5.7–11.6)	1

^1^ Age-adjusted seroprevalence in the Israeli standard population as in “lamas”, 2017 (15–19, 20–24, 25–34, 35–44, 45–54, 55–64, 65–74, 75+). ^2^ Reference group.

**Table 4:** Associations between study characteristic variables and HEV IgG.

Variables		Negative IgG HEV N = 641	Positive IgG HEV N = 75	Univariable Models OR (95% CI)	Multivariable Model OR (95% CI)
Sex	Male	276 (43.1)	30 (40.0)	1	1
	Female	365 (56.9)	45 (60.0)	1.13 (0.69–1.85)	1.23 (0.74–2.12)
Age (years)	18–34	253 (39.4)	7 (9.3)	1	1
	35–54	249 38.9)	14 18.7)	2.03 (0.81–5.12)	2.46 (0.96–6.30)
	55–74	114 (17.8)	31 (41.3)	9.83 (4.20–22.98)	14.38 (5.87–35.25)
	75+	25 (3.9)	23 (30.7)	33.25 (12.98–85.17)	59.05 (21.11–165.13)
Ethnicity	Arab	293 (45.7)	42 (56.0)	1	1
	Jewish	348 (54.3)	33 (44.0)	0.66 (0.41–1.07)	0.57 (0.26–1.24)
Place of residence	Village	274 (42.8)	40 (53.3)	1	1
	City	367 (57.2)	35 (46.7)	0.65 (0.40–1.06)	0.96 (0.46–1.99)
Socioeconomic status	Low rank 1–3	160 (24.9)	24 (32.0)	1	1

## Data Availability

Data is unavailable due to privacy restrictions.
